# A Long-Term Retrospective Natural History Study of *EFEMP1*-Associated Autosomal Dominant Drusen

**DOI:** 10.1167/iovs.65.6.31

**Published:** 2024-06-20

**Authors:** Thales A. C. de Guimarães, Angelos Kalitzeos, Omar A. Mahroo, Jacqueline van der Spuy, Andrew R. Webster, Michel Michaelides

**Affiliations:** 1 UCL Institute of Ophthalmology, University College London, London, United Kingdom; 2 Moorfields Eye Hospital NHS Foundation Trust, London, United Kingdom

**Keywords:** *EFEMP1*, drusen, macular degeneration, optical coherence tomography, natural history, anti-angiogenics, choroidal neovascularization, fundus autofluorescence, dominant drusen

## Abstract

**Purpose:**

To analyze the natural history of *EFEMP1*-associated autosomal dominant drusen (ADD).

**Methods:**

In this retrospective observational study of molecularly confirmed patients with ADD, data and retinal imaging were extracted from an in-house database. The main outcome measurements were best-corrected visual acuity (BCVA), refraction, and retinal imaging, including quantitative analyses of the outer nuclear layer (ONL) thickness and pigmented epithelium detachment area, as well as qualitative analyses.

**Results:**

The study included 44 patients (34 females and 10 males). The mean ± SD age of symptom onset was 40.1 ± 6.59 years of age (range, 25–52). Fourteen patients were asymptomatic during their entire follow-up. The most common symptoms at presentation were reduced vision (70%) and distortion in central vision (53%). Most subjects were emmetropic. The mean BCVA (logMAR) at baseline was 0.27 ± 0.41 (range, −0.1 to 2.1) in right eyes and was 0.19 ± 0.32 (range, −0.2 to 1.3) in left eyes. After a mean follow-up of 7.9 years, BCVA was reduced to 0.59 ± 0.66 (range, −0.1 to 2.1) in right eyes and 0.5 ± 0.72 (range, −0.1 to 2.4) in left eyes, values that were significantly different than baseline (*P* < 0.0001 and *P* < 0.0014, respectively). Fifteen eyes showed active or inactive choroidal neovascularization (CNV). BCVA differed significantly (*P* = 0.0004) between eyes with and without CNV at a comparable mean age. The ONL had a slow rate of thinning longitudinally, which significantly correlated with BCVA.

**Conclusions:**

Despite the late onset and relatively good prognosis of ADD, CNVs are more frequent than previously reported and are associated with a worse prognosis. Further research is necessary to elucidate gender associations.

Autosomal dominant drusen (ADD) is an inherited retinal dystrophy (IRD) that was first described in 1899. It is also known as Doyne honeycomb retinal dystrophy or Malattia Leventinese. It was mapped to a region on chromosome 2p16, with a c.1033C>T p.(R345W) variant being subsequently identified in the fibulin-3 gene, *EFEMP1* (OMIM *601548).^1^^–^^3^ A series of R345 mutants and a cell-based luminescence assay developed by Hulleman et al.^4^ suggest that aromatic residue substitutions at position 345 cause significant secretion deficiencies in *EFEMP1*, which may be mediated by reduced disulfide bonding in domain 6. In a knockout mouse model, another group provided evidence that this substitution results in the accumulation of membranous deposits below the retinal pigment epithelium (RPE).^5^

ADD is characterized by early-onset drusen deposits, which may be distributed in a radial pattern and can often be found around the edge or abutting the optic nerve head.^6^ These deposits are external to the RPE, occupying the entire thickness of Bruch's membrane, which causes progressive RPE atrophy of the posterior pole and can be complicated by choroidal neovascularization (CNV).^1^^,^^7^^,^^8^

The largest cohort of molecularly confirmed individuals to date was published by Michaelides et al. in 2006.^7^ Herein, we aim to expand on that report and present a long-term detailed description of the clinical course and retinal imaging in a cohort of 44 patients. Combined, these data are vital for genetic counseling, monitoring, and prognostication purposes, while also providing a crucial step toward the design of prospective natural history studies and development of future clinical trials.

## Materials and Methods

This study adhered to the tenets of the Declaration of Helsinki and was approved by the local Ethics Committee (REC 12/LO/0141). All patients were seen in a single center (Moorfields Eye Hospital, London, UK). Informed consent was obtained from all patients.

### Patient Identification

Inclusion criteria for this study was molecular confirmation of the R345W variant in *EFEMP1* and phenotypic confirmation of ADD. All subjects were identified in the genetics database of Moorfields Eye Hospital.

### Molecular Diagnosis

A combination of next-generation and direct Sanger sequencing, including panels of retinal dystrophy genes, was used to identify variants in *EFEMP1*.

### Clinical Assessment

All patients were seen by inherited retinal disease specialists at Moorfields Eye Hospital. Clinical notes were thoroughly reviewed, and data from best-corrected visual acuity (BCVA), refraction, fundoscopy, and slit-lamp examination were gathered. BCVA and refraction evaluations were performed by an optometrist.

### Retinal Imaging

Fundus autofluorescence (FAF) and optical coherence tomography (OCT) were performed using SPECTRALIS OCT (Heidelberg Engineering, Heidelberg, Germany), and widefield retinal imaging was obtained with an Optos device (Optos, Dunfermline, UK). All available data were analyzed cross-sectionally and longitudinally.

### FAF Analysis

The FAF images were qualitatively assessed for the pattern of autofluorescence (AF). No quantitative analysis was performed.

### OCT Analysis

**Figure 1.**
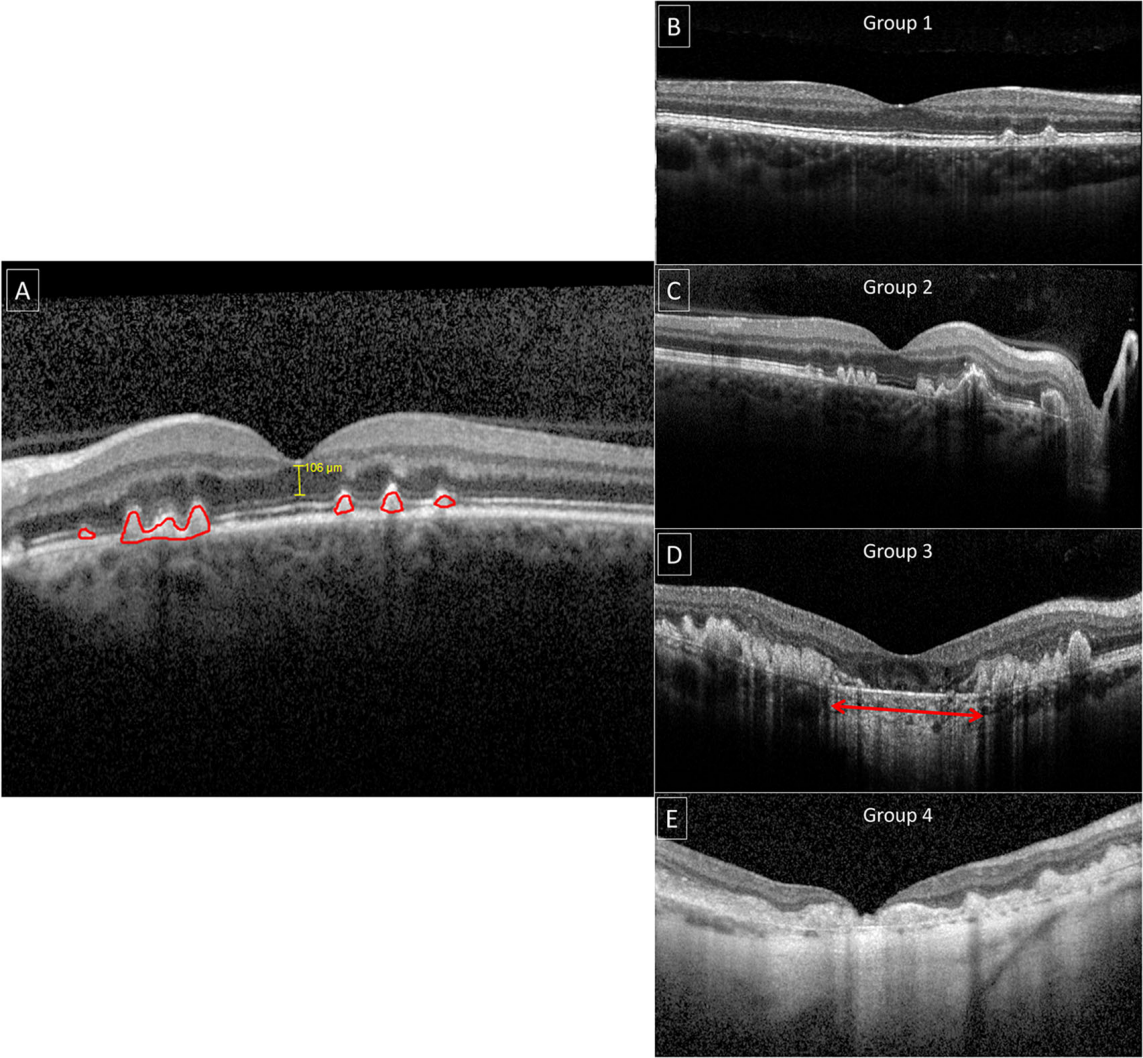
Quantitative and qualitative analyses of spectral-domain optical coherence tomography (SD-OCT). (**A**) ONL thickness (*yellow*) and PED area (*red*) were measured longitudinally as shown. For accuracy, all measurements were made on a 1 × 1-µm scale. (**B**–**E**) Qualitative OCT grading (OCT groups 1–4, respectively). The *red* *double-arrowed line* in **D** shows an area of RPE atrophy with a corresponding choroidal hyper transmission defect.

OCT was analyzed qualitatively according to the presence of specific features, with patterns being identified on the cross-sectional and longitudinal B-scans. We grouped patients into four OCT groups based on the features identified in the transfoveal scan: (1) presence of well-demarcated drusenoid pigmented epithelium detachment (PED) but preserved outer retinal/RPE complex architecture; (2) presence of coalescent PED and signs of outer retinal structural loss, such as ellipsoid zone disruption or outer retinal thinning with a preserved RPE; (3) presence of PED, outer retinal structural loss, and localized RPE atrophy with or without active CNV; and (4) diffuse RPE atrophy and/or presence of fibrotic tissue/disciform scar with or without active CNV (Fig. 1).

Quantitative analysis was obtained using the digital calipers provided in HEYEX Version 2.5.5 (Heidelberg Engineering). The measurements were made in the transfoveal horizontal line scan at maximum magnification in a 1 × 1-µm display to reduce inaccuracies, with the foveal reflex used as anatomical reference. The outer nuclear layer (ONL) thickness was measured in the transfoveal B-scan of patients that had discernible outer retinal architecture as the distance between the internal and external limiting membrane at the foveal center.^9^^,^^10^ Similarly, the PED area (for individuals with OCT groups 1–3) was measured manually in the single transfoveal B-scan and summed with the area tool also provided by the HEYEX software, as shown in Figure 1. Follow-up mode was used in all patients. The OCT quantitative parameters were compared with BCVA to explore structure–function correlations.

### Statistical Methods

Statistical analysis was performed with the aid of Prism 9 (GraphPad, Boston, MA, USA). Parametric and non-parametric tests were employed, as well as correlation parameters (either Pearson or Spearman). A Kaplan–Meier survival analysis was performed where the outcome event was a vision of 6/60 (1.0 logMAR), the definition of sight impairment by the Royal National Institute of Blind People. Significance of all statistical tests was set at *P* < 0.05, and the D'Agostino–Pearson test (omnibus *K*^2^) was used to determine normality for all variables. Descriptive statistics were used to describe all quantitative parameters, followed by range, standard deviation (SD), or standard error of the mean (SEM).

## Results

### Demographics and Symptoms

Forty-four subjects were identified with the R345W variant in *EFEMP1*, all of which had a clinical diagnosis compatible with ADD. Our cohort was comprised of 34 female patients (77%) and 10 male patients (23%), and all patients were seen at least once. The mean ± SD age at the baseline visit was 45.3 ± 11.9 years (range, 21–69). Follow-up data were available for 35 patients, with a mean follow-up time of 7.9 ± 5.1 years (range, 1–23). The mean age of symptom onset was 40.1 ± 6.5 years (range, 25–52); 14 patients were asymptomatic during their entire follow-up period. The most commonly reported symptoms at onset were reduced vision (70%) and mild distortion in central vision (53%). Two patients developed nyctalopia, and another one developed photophobia in their late 50s. Interestingly, one patient had a concurrent diagnosis of oculocutaneous albinism (with foveal hypoplasia) and had nystagmus and reduced central vision from birth. This patient was not found to have any variants in albinism-related genes on a large panel of IRD-related genes.

### Refraction and Clinical Features

**Figure 2.**
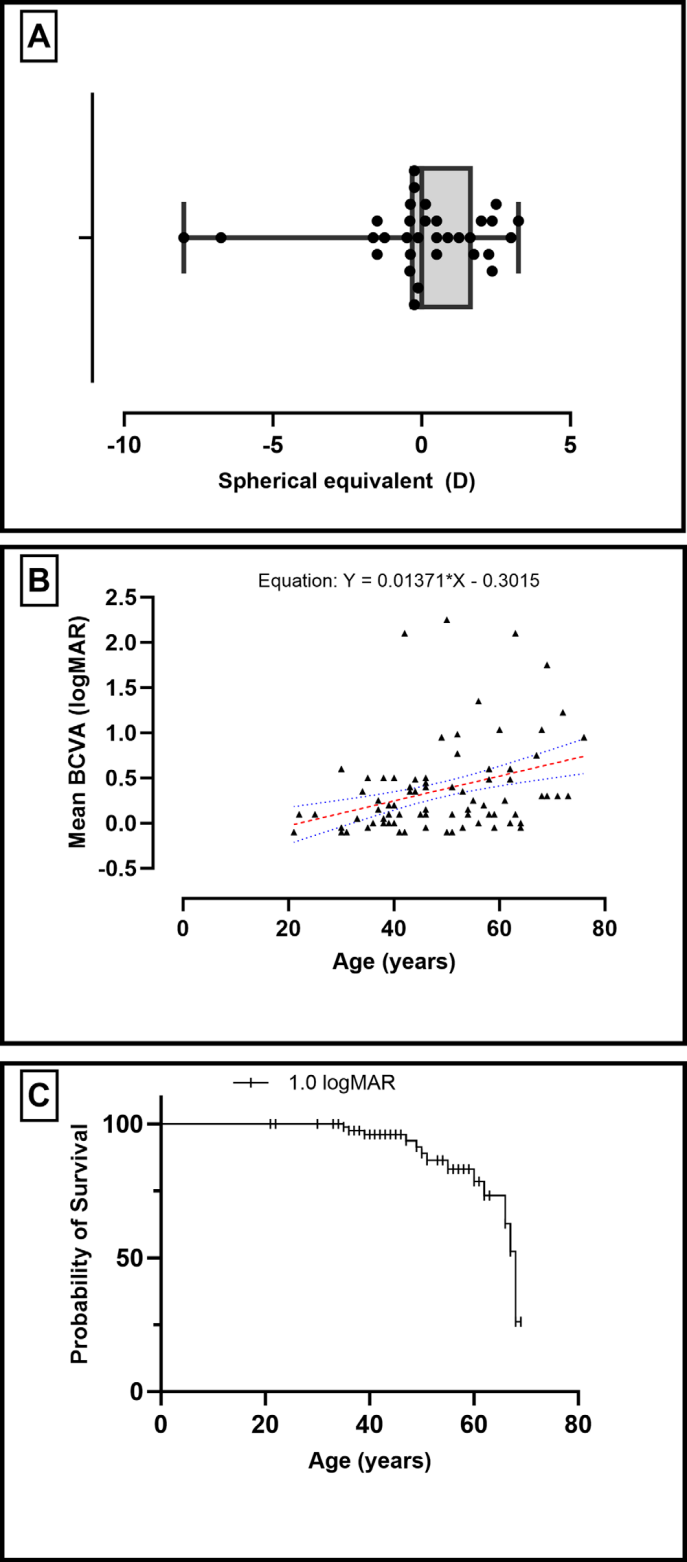
Correlation and survival of BCVA against age. (**A**) Box-and-whisker plot showing the median spherical equivalent, quartiles, and range (with values represented by *circles*). (**B**) Simple linear regression of the mean BCVA against age. The *red dashed line* is the best-fit, whereas the *blue dotted curves* represent the 95% confidence interval. (**C**) A simple survival analysis (Kaplan–Meier) reviews the probability of survival (with the outcome event set as BCVA worse or equal to 1.0 logMAR) in each age group.

Most subjects were emmetropic (Fig. 2A). The mean ± SD spherical equivalent was 0.04 ± 2.3 D (range, −8.0 to 3.25). The mean BCVA values at the baseline visit were 0.27 ± 0.41 logMAR (range, −0.1 to 2.1) in right eyes and 0.19 ± 0.32 logMAR (range, −0.2 to 1.3) in left eyes, which was significantly correlated (*r* = 0.59; *P* < 0.0001, Spearman correlation) and not significantly different (*P* = 0.2, Wilcoxon matched-pairs signed rank test). In the 35 patients for whom follow-up was available, the mean BCVA values at the last visit (at a mean follow-up time of 7.9 years) were 0.59 ± 0.66 (range, −0.1 to 2.1) in right eyes and 0.5 ± 0.72 (range, −0.1 to 2.4) in left eyes, both of which were significantly different than baseline (*P* < 0.0001 and *P* < 0.0014, respectively; Wilcoxon matched-pairs signed rank test). A simple linear regression was plotted of age against BCVA (logMAR) in right and left eyes, revealing a direct relationship with a significantly non-zero slope (*F* = 9.7 and 7.98, *P* = 0.0025 and 0.006, respectively). The mean BCVA values of both eyes were also plotted against age and was found to be also significant (*F* = 17.8; *P* < 0.0001) (Fig. 2B). Finally, a simple survival analysis (Kaplan–Meier) showed that 78% of the cohort had BCVA better than 1.0 logMAR at the age of 60 years (Fig. 2C).

On examination, the anterior segment was normal in all cases, apart from four cases (9%) with age-related nuclear cataract diagnosed at a mean age of 61 years (range, 51–66). The fundus findings were typical as reported in detail in another study.^7^ They revealed yellow–white drusenoid deposits at a wide variety of sizes and numbers. Some individuals had confluent macular drusen with or without areas of RPE atrophy, whereas others had well-defined radial drusen. No abnormalities in the peripheral retina were observed in any subject. One interesting finding in our cohort was the presence of disciform scars indicative of previous CNV in both eyes of two patients (13%), which will be discussed below.

### Choroidal Neovascularization

**Figure 3.**
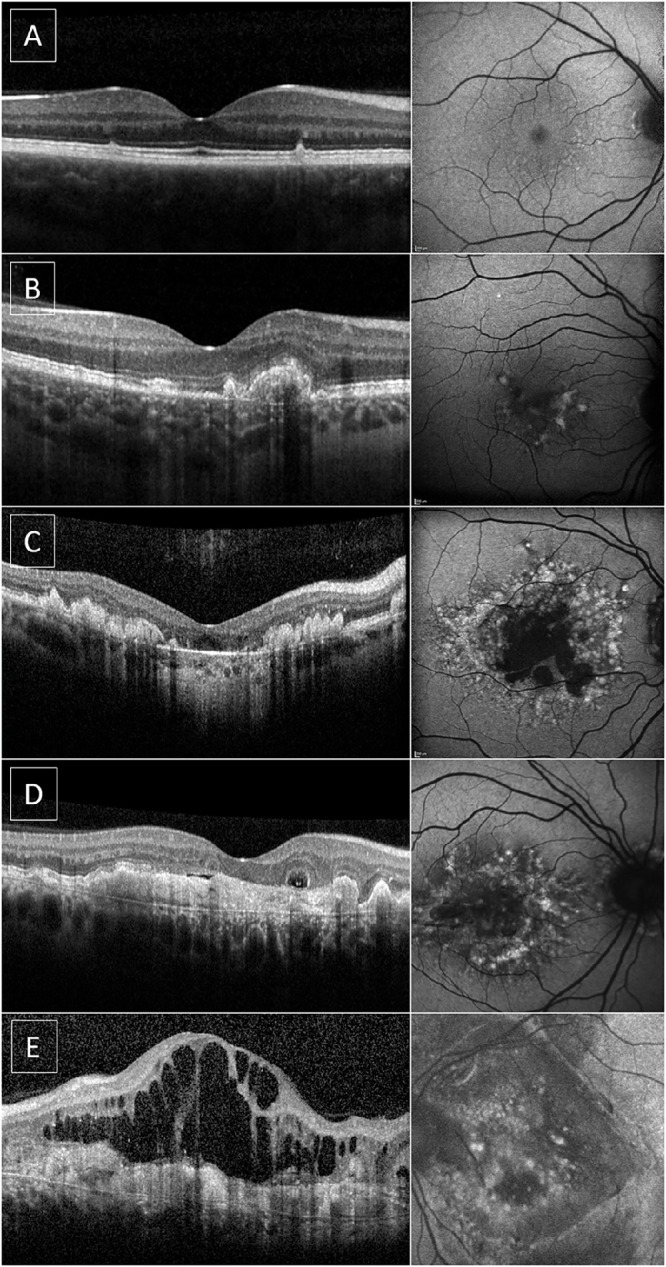
Multimodal imaging of patients in different disease stages by SD-OCT and short-wavelength FAF of affected individuals, each row corresponding to one patient. (**A**, **B**) Discrete, well-delimited drusen are visible in both imaging modalities (**A**), which may eventually enlarge and coalesce, as for the patient in the second row (**B**). (**C**) Another subject with a transfoveal B-scan appearance compatible with OCT group 3, with coalesced drusen, outer retinal thinning, and focal RPE atrophy with a hypertransmission defect, which corresponds to a definitely decreased area of autofluorescence in the FAF. (**D**) Presence of subretinal fibrotic tissue with a small amount of subretinal fluid, suggestive of active CNV. An outer retinal tubulation is also present. (**E**) Large amount of intraretinal fluid in a patient undergoing treatment with anti-angiogenics for CNV (bevacizumab). There is a considerable amount of intraretinal fluid on top of a thick subretinal fibrosis, the latter of which corresponds to the disciform scar seen in the FAF.

**Figure 4.**
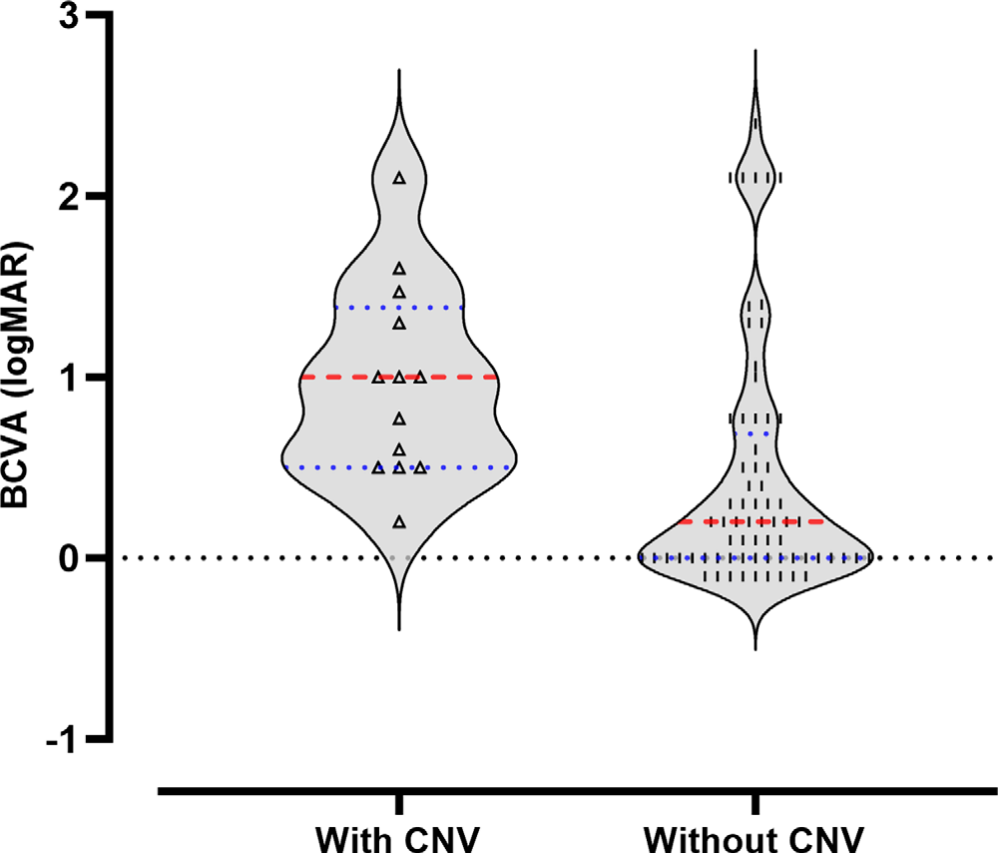
Comparison of BCVA in eyes with and without CNV. Violin plot shows the median (*red dashed lines*), quartiles (*blue dotted lines*), and overall distribution of all eyes with CNV (*triangle symbols*) and without CNV (*vertical trace symbols*).

Fifteen eyes of 12 patients (27%) had active scars (*n* = 10 patients) or disciform scars (*n* = 2 patients) that were indicative of previous CNV (Fig. 3), which is a considerably higher prevalence than previously reported.^7^ Three patients (30%) in the active subgroup had bilateral CNV. The mean age at the CNV onset was 48.4 years (range, 36–64; 95% confidence interval, 42.2–54.7). The mean ± SD BCVA at the last visit of eyes with CNV was 0.96 ± 0.54 logMAR (range, 0.2–2.1) at a mean age of 55.1 ± 10.9 years (range, 38–69), and it was 0.44 ± 0.66 (range, −0.1 to 2.4) for eyes without CNV at a mean age of 51.9 ± 13.5 (range, 25–76) (Fig. 4). BCVA differed significantly (*P* = 0.0004, Mann–Whitney test) between eyes with and without CNV at a comparable mean age (*P* = 0.41, unpaired *t*-test with Welch's correction).

All patients identified with active CNV were treated with intravitreal injections of anti-angiogenic drugs, albeit in different tertiary hospitals due to geographical restrictions and difficulty with attending our site; hence, the subsequent data are incomplete and should be considered descriptive. Information regarding the total number of injections up until the last visit was available for 11 eyes of nine patients with CNV. The mean ± SD number of injections per eye was 5.9 ± 5.9 (range, 2–22) during the follow-up; four eyes in which the CNV remained active are still under treatment after a mean time of 5.5 ± 3.5 years.

### Retinal Imaging

Thirty-nine patients had at least one FAF and OCT at a baseline mean ± SD age of 46.4 ± 13 years (range, 21–75), whereas longitudinal retinal imaging was available for 33 individuals at a mean age of 53.7 ± 13.2 years (range, 26–75) with a mean follow-up of 6.6 ± 3.8 years. Figure 3 illustrates patients at different disease stages.

#### Fundus Autofluorescence

**Figure 5.**
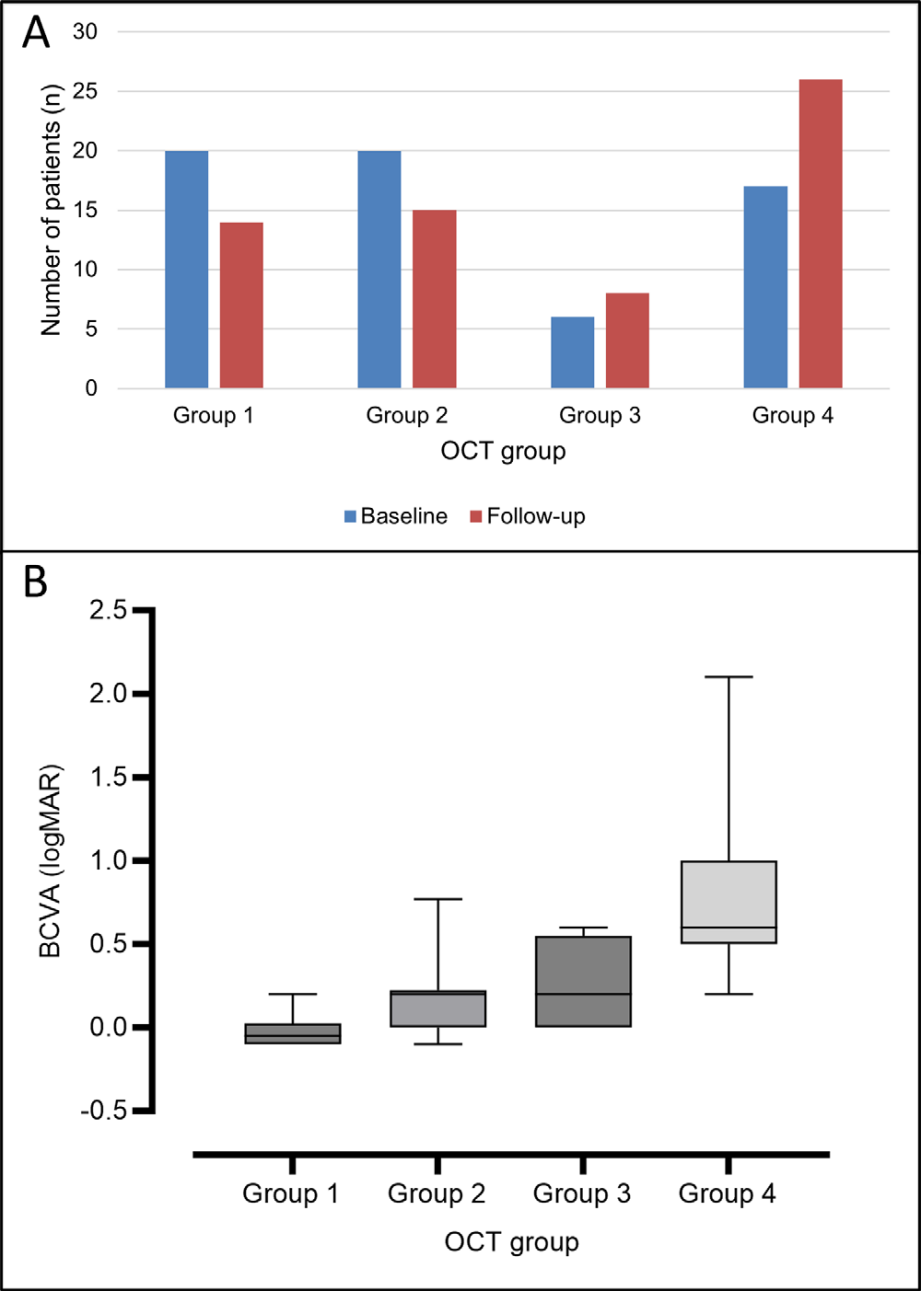
OCT data analysis. (**A**) Number of patients in each OCT group at baseline and follow-up. (**B**) Box-and-whisker plots illustrate the median BCVA, quartiles, and range in each OCT group. The BCVA was significantly worse in individuals in group 4.

Drusen were present in all subjects and corresponded to areas of increased AF signal in the majority of cases, albeit not all drusen seen in the fundus were associated with increased AF, a feature previously reported by our group.^7^^,^^11^ Centrally decreased autofluorescence areas were present in eight eyes of four patients, corresponding with deep retina-RPE complex atrophy identified on OCT and color fundus photography (Fig. 5). Two patients also had optic nerve drusen.

#### Optical Coherence Tomography

Patients were grouped as described in the methodology. At baseline, 26 eyes (33.8%) were in group 1, 22 eyes (28.6%) were in group 2, nine eyes (11.7%) were in group 3, and 20 eyes (25.9%) were in group 4. During follow-up, 22 eyes changed groups (Fig. 5A). Twenty-nine patients (74.3%) had symmetric findings at baseline and were classified in the same group, with the biggest disparity being individual MEH005, who had a disciform scar in the right eye and small, well-defined PED in the left. Further, two patients with late-stage disease had outer retinal tubulations (Fig. 3), a feature previously reported in age-related macular degeneration (AMD) and in certain inherited retinal disorders, such as choroideremia.^12^^–^^14^

Quantitative analysis was then performed. For context, the mean ± SD ONL thickness in a normative population composed of 200 adults was 92.2 ± 9.3 µm.^15^ The mean ONL thickness at baseline was 83 ± 30.4 µm (range, 0–124) in right eyes and 84 ± 33.5 µm (range, 0–136) in left eyes, which was significantly correlated (*r* = 0.77; *P* < 0.0001, Spearman correlation). After a mean follow-up of 6.6 ± 3.8 years, the ONL thickness reduced to 64.5 ± 32.1 µm in right eyes and 67.8 ± 37.8 µm in left eyes, which was significantly different (*P* < 0.0001 for both eyes, Wilcoxon matched-pairs signed-rank test). This difference, albeit statistically significant, has reduced clinical significance given it is below the 4-µm axial resolution of the spectral-domain optical coherence tomography device used herein.

The cross-sectional PED area in the transfoveal B-scan was also measured in individuals with OCT groups 1 to 3, because, by definition, group 4 had subretinal fibrosis/disciform scars. Fifty-one eyes of 27 patients had a measurable PED at a mean baseline age of 44 ± 12.3 years (range, 21–65), with a mean PED area of 0.21 ± 0.17 µm^2^ (range, 0.01–0.68) in right eyes and 0.17 ± 0.15 µm^2^ (range, 0.01–0.57) in left eyes, which was significantly correlated in patients with bilateral PED (*r* = 0.77; *P* < 0.0001, Pearson correlation coefficient). After a mean follow-up of 9.4 ± 3.5 years, the mean PED changed to 0.24 ± 0.18 µm^2^ (range, 0.01–0.7) in right eyes and 0.24 ± 0.2 µm^2^ (range, 0.01–0.68) in left eyes, both of which failed to achieve statistical significance (*P* = 0.53 and *P* = 0.22 for right and left eyes, respectively; unpaired *t*-test with Welch's correction). The longitudinal data for PED should be considered descriptive due to a considerable proportion of patients having changes in the OCT grouping.

### Structure–Function Correlations

Eyes that were classified as OCT group 4 had an overall lower BCVA than individuals with more preserved retinal architecture (Fig. 5B). The mean ± SD logMAR BCVA measurements from groups 1 to 4 were, respectively, −0.01 ± 0.11, 0.18 ± 0.21, 0.24 ± 0.25, and 0.67 ± 0.45, which were significantly different (*P* < 0.0001, Kruskal–Wallis one-way ANOVA). The biggest difference between BCVA values was between groups 1 and 4, with a difference between means (±SEM; *P*; unpaired *t*-test with Welch's correction) of 0.68 LogMAR (±0.11; <0.0001); whereas there was no statistically significant difference between BCVA in groups 2 and 3 (±0.1; 0.44). Furthermore, there was a significant difference between the mean BCVA of groups 1 and 2, with a difference between means of 0.2 logMAR, which was significant (*P* = 0.0003), thereby highlighting the absence of functional deficits in group 1 even when compared to group 2, the second mildest group from a structural and functional perspective.

The structural parameters derived from OCT at baseline were then correlated with BCVA. ONL thickness and BCVA (logMAR) had an inverse moderate correlation that was statistically significant (*r* = −0.44; *P* < 0.0001, Spearman correlation). PED area in the transfoveal scan had a weakly positive correlation that did not quite reach statistical significance (*r* = 0.25; *P* = 0.051, Spearman correlation). BCVA in the present work is expressed in logMAR; hence, the above numbers imply progressively worse vision with thinner ONLs and with larger PEDs in the transfoveal scan, both of which are consistent with clinical expectations.

## Discussion

We investigated the retinal phenotype of ADD, both cross-sectionally and longitudinally. This represents the largest cohort of affected patients reported to date. The retinal phenotype has been well described in previous reports and is in keeping with our findings.^7^ We were able to broadly characterize the clinical and retinal imaging features. An unexpected finding was an apparent sex imbalance in our cohort, with a male-to-female ratio of 1:3.4, which has been recently reported by our group in a range of genotypes, including *EFEMP1*.^24^ Interestingly, some studies in AMD (to which *EFEMP1* overexpression may contribute to neovascularisation^16^) have pointed out a higher likelihood in women of early AMD, and a potentially higher burden and risk for the neovascular form of the disease if compared to men.^17^^–^^19^ The reason of this apparent imbalance in the present cohort is unclear and may have been subject to selection bias.

From a clinical perspective, ADD remains a relatively late-onset disease with symptoms starting typically in the 40s, most of which are mild, such as central vision distortion, albeit 70% of the cohort presented with decreased vision. Our data suggest that ADD is not associated with any specific type of refractive errors. Similarly, most patients maintain functional vision throughout the disease course. Clinically, two unrelated patients had optic nerve drusen, which are likely to be incidental findings. Surprising, though, was the relatively high prevalence of CNV formation, which was much higher than previously reported.^7^ Reactivation of CNV can happen even in late-stage disease with concurrent subretinal fibrotic tissue. Although these data are incomplete, given the uncertain number and frequency of anti-angiogenic injections, there were significantly fewer injections performed than would be expected for the neovascular form of AMD, which could suggest a milder behavior of the CNV associated with ADD. The presence of CNV is associated with a significantly worse natural history and overall vision loss. In light of this, we recommend regular follow-up of affected individuals (e.g., annually), as well as adequate patient counseling with thorough explanation of symptoms suggestive of CNV and an immediate visit to an eye service unit.

We performed in-depth retinal imaging, which is imperative for patient prognostication. The qualitative classification used in this study does carry some degree of subjectiveness. Therefore, for example, grouping OCTs of affected individuals is to be taken as a “soft” recommendation, albeit it does help in distinguishing disease stages, particularly given its direct association with BCVA. Similarly, quantitative analyses of OCTs are not straightforward in the context of ADD. Establishing measurements of PED area in early disease phases (i.e., OCT group 1) is an achievable task, which tends to become progressively more difficult, particularly when the drusen begin to coalesce and in the presence of concurrent outer retinal/RPE abnormalities. Nevertheless, with the advances in imaging device capabilities, such as follow-up mode—allowing the exact same retinal loci to be imaged longitudinally—and faster scanning speeds (OCT2, 85 kHz), it is feasible to quantify with accuracy, at least, the ONL thickness, which is inversely correlated with BCVA as shown in our results. Indeed, the structural retinal changes appear to follow a specific natural history pattern: (1) well-defined PED, which (2) coalesce and eventually lead to loss of outer retinal lamination and subretinal fibrosis, after which (3) RPE atrophy develops.

Future prospective studies would be essential to further establish ideal prognostic measurements and endpoints for future clinical trials. The universal presence of the point variant R345W in affected individuals opens up avenues for treatment such as, for example, (non-vector delivered) gene editing. Given the chorioretinal pathogenesis of ADD, it is not unfeasible that suprachoroidal delivery of gene therapy products could provide an attractive route for a more posterior and circumferential distribution of investigational medical products.^20^^,^^21^ It remains to be established what benefits patients might experience with therapy, given the relatively slow disease progression and relatively good prognosis. The use of high-resolution imaging techniques, such as adaptive optics scanning light ophthalmoscopy,^22^ and devices to assess retinal sensitivity in detail, such as fundus-guided perimetry,^23^ may provide further insights into the structural and loci-specific functional integrity of the photoreceptor mosaic, ideally in prospective, robust, long-term natural history studies.

This study has limitations, particularly given its retrospective nature. Follow-up intervals and protocols for BCVA acquisition were not standardized, nor were the imaging techniques (i.e.,. FAF and OCT were unavailable in some visits). Moreover, no further functional data were available, which remains a gap that must be addressed.

## Conclusions

This report of molecularly confirmed individuals with ADD helps to further define the phenotypic spectrum. This condition is an adult-onset dystrophy characterized by the presence of drusen most typically in individuals around 40 years of age. Most subjects in this cohort were female, although the significance of this is unclear. Watchful follow-up of affected patients is advisable, as CNVs are more common than previously thought, which significantly worsens the prognosis and the natural history of the disease.
